# P-2124. Validation of BM-AspICU Clinical Algorithm in Critically Ill Patients with Suspected Invasive Pulmonary Aspergillosis in Medical ICU Using Post-Mortem Minimally Invasive Tissue Sampling

**DOI:** 10.1093/ofid/ofae631.2280

**Published:** 2025-01-29

**Authors:** Anuj Ajayababu, Animesh Ray, Gagandeep Singh, Immaculata Xess

**Affiliations:** ALL INDIA INSTITUTE OF MEDICAL SCIENCES NEW DELHI INDIA, NEW DELHI, Delhi, India; All India Institute of Medical Sciences, New Delhi, India, New Delhi, Delhi, India; All India Institute of Medical Sciences, New Delhi, New Delhi, Delhi, India; All India Institute of Medical Sciences, New Delhi, New Delhi, Delhi, India

## Abstract

**Background:**

Post-mortem studies have shown significant underestimation of invasive pulmonary aspergillosis in critically ill ICU patients, in resource-limited settings due to limited awareness, diagnostic capabilities, and ubiquitous presence of *Aspergillus* spores. The revised BM AspICU algorithm for critically ill patients was validated in a historical cohort, with changes in the host and imaging criteria as well as inclusion of PCR assays. This study for the first time prospectively evaluated the utility of the criteria in a cohort of ICU patients, using post-mortem minimally invasive tissue sampling.

**Figure 1.**
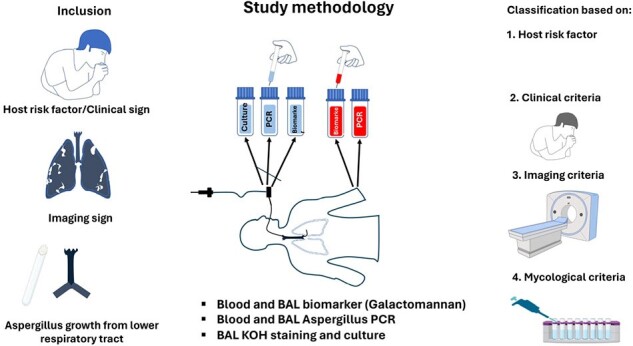
Study methodology (Prospective validation of BM Asp ICU clinical algorithm)

Patients on mechanical ventilation with a host risk factor, clinical or imaging sign or Aspergillus growth from lower respiratory tract were recruited as per the BM-Asp ICU algorithm . All patients underwent blood and bronchoalveolar lavage fluid Aspergillus PCR on Day 0, 6 and 12 and biomarker (galactomannan) estimation in blood and lavage fluid every third day till death/recovery from illness. The analysis was done on patients who expired and post-mortem minimally invasive tissue samples could be obtained for PCR, KOH staining, and culture and histopathology.

**Methods:**

Patients on mechanical ventilation with a host risk factor, clinical or imaging sign or *Aspergillus* growth in lower respiratory tract were recruited (Figure 1). All patients underwent blood and bronchoalveolar lavage fluid *Aspergillus* PCR and biomarker (Galactomannan) estimation in blood and lavage fluid till death/recovery from illness. Post-mortem MITS was done to obtain samples for histopathology, KOH staining for hyphae and culture and *Aspergillus* PCR to enable classification as definite IPA/not as per the revised algorithm.

**Figure 2:**
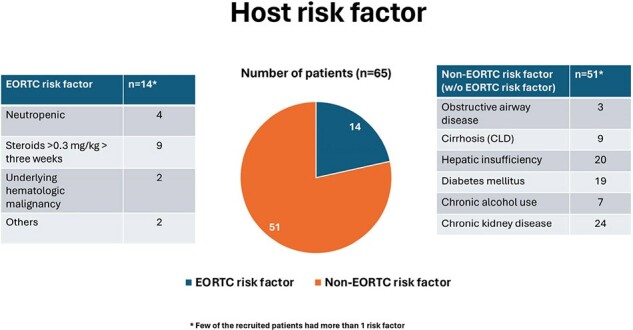
Host risk factors in study population

**Results:**

A total of 135 patients were included, out of whom 99 expired. MITS was done in 66 patients, allowing classification as definite IPA or not. The mean age was 43+16 years with 47% males (n=66). 21% (n=14) had an EORTC risk factor while of the remaining, 98% (n=51) had a non-EORTC risk factor for IPA (Figure 2). The overall tests characteristics for the BM Asp ICU clinical algorithm showed a sensitivity of 84.5% [95% CI (72.6-92.6)], specificity of 42.9% [95% CI (9.9-81.6)], PPV of 92.4% [95% CI (86.5-95.9)] and NPV of 25% [95% CI (10.5-48.7)]. The test performance of biomarker compared poorly when compared to PCR in blood and BAL samples (Figure 3).

**Figure 3:**
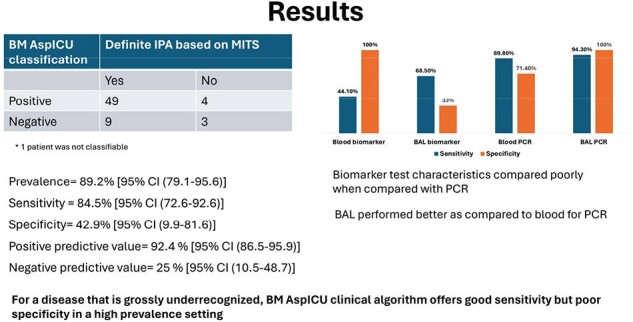
Study results

**Conclusion:**

The revised BM Asp ICU clinical algorithm caters to non-neutropenic critically ill patients in ICU, under-recognised by the EORTC MSG criteria. This first-of-its-kind prospective study shows moderate sensitivity in a high prevalence setting, indicating a possible need for further modifications to ensure timely recognition of IPA. Biomarkers compared poorly when compared to PCR overall, with BAL PCR performing better as compared PCR testing in blood samples.

**Disclosures:**

All Authors: No reported disclosures

